# Work and health among immigrants and native Swedes 1990–2008: a register-based study on hospitalization for common potentially work-related disorders, disability pension and mortality

**DOI:** 10.1186/1471-2458-12-845

**Published:** 2012-10-05

**Authors:** Bo Johansson, Magnus Helgesson, Ingvar Lundberg, Tobias Nordquist, Ola Leijon, Per Lindberg, Eva Vingård

**Affiliations:** 1 Department of Medical Sciences, Occupational and Environmental Medicine, Ulleråkersvägen 38-40, Uppsala, SE-751 85, Sweden; 2 Occupational and Environmental Medicine, Uppsala University Hospital, Uppsala University, Ulleråkersvägen 38-40, Uppsala, SE-751 85, Sweden; 3 Department of Public Health Sciences, Division of Occupational and Environmental Medicine, Karolinska Institutet, Norrbacka, Stockholm, SE-171 76, Sweden; 4 The Swedish Social Insurance Inspectorate, Fleminggatan 7, Box 202, Stockholm, SE-101 24, Sweden; 5 Department of Occupational and Public Health Science, Centre for Musculoskeletal Research, University of Gävle, Kungsbäcksvägen 47, Gävle, SE-80176, Sweden

**Keywords:** Immigrant, Migration, Health, Hospitalization, Disability pension, Mortality, Labor market, Employment, Unemployment, Sweden

## Abstract

**Background:**

There are many immigrants in the Swedish workforce, but knowledge of their general and work-related health is limited. The aim of this register-based study was to explore whether documented migrant residents in Sweden have a different health status regarding receipt of a disability pension, mortality and hospitalization for lung, heart, psychiatric, and musculoskeletal disorders compared with the native population, and if there were variations in relation to sex, geographical origin, position on the labor market, and time since first immigration.

**Methods:**

This study included migrants to Sweden since 1960 who were 28–47 years old in 1990, and included 243 860 individuals. The comparison group comprised a random sample of 859 653 native Swedes. These cohorts were followed from 1991 to 2008 in national registers. The immigrants were divided into four groups based on geographic origin. Hazard ratios for men and women from different geographic origins and with different employment status were analyzed separately for the six outcomes, with adjustment for age, education level, and income. The influence of length of residence in Sweden was analyzed separately.

**Results:**

Nordic immigrants had increased risks for all investigated outcomes while most other groups had equal or lower risks for those outcomes than the Swedes. The lowest HRs were found in the EU 15+ group (from western Europe, North America, Australia and New Zealand). All groups, except Nordic immigrants, had lower risk of mortality, but all had higher risk of disability pension receipt compared with native Swedes. Unemployed non-Nordic men displayed equal or lower HRs for most outcomes, except disability pension receipt, compared with unemployed Swedish men. A longer time since first immigration improved the health status of men, while women showed opposite results.

**Conclusions:**

Employment status and length of residence are important factors for health. The contradictory results of low mortality and high disability pension risks need more attention. There is great potential to increase the knowledge in this field in Sweden, because of the high quality registers.

## Background

Immigrants are often depicted as a vulnerable group in terms of exclusion from the labor market, and the participation of migrants in the Swedish labor market has been well researched since the 1970s. Several studies have presented in detail the historic immigration from the extensive post-World War II labor migration, predominantly from other Nordic countries and Southern and Eastern Europe, through the increasingly restrictive policy following the oil crisis in the early 1970s, to the mainly refugee and family reunification immigration in recent decades, dominated by migrants of non-European and former Yugoslavian origin. These studies also described the changing regulative policies [1-3]. However, far less has been studied on the health status and health transformations of these groups, and there is very fragmentary knowledge of health related to immigration and labor market participation in Sweden as well as internationally.

The international literature supports the view that empiric data are too meager to allow any comparative analysis between immigrants and natives concerning work-related health [4-7]. However, there are reasons to believe that work-related health among the immigrant population differs from that of the native populace in various countries around the world [8-11], in Europe [12] and in Sweden [13-16]. It may also vary in relation to gender, the reason for migration, geographical origin, age at migration, and time in the new country of residence.

It has been demonstrated that immigrants to Sweden suffer more from physical as well as psychological problems than native Swedes, although there are differences with regard to diagnoses and the origin of the migrants [14-16]. The literature on migrant health has drawn attention to the importance of the length of time spent in the new country. Research shows contradicting results regarding the changes in health status of immigrants over time in the new country of residence. Some studies of specific migrant groups and/or specific diagnoses have reported an improvement in health [17,18], while others found the opposite [19,20]. More general studies displayed more complex features, with variations for different immigrant groups and different diagnoses [21-23].

The aim of this longitudinal study was to explore whether immigrants of active working age had a different health status concerning disability pension, mortality, and hospitalization for lung-, heart-, psychiatric-, and musculoskeletal disorders in comparison with the native Swedish population. It also explored whether the health status differed in relation to sex, geographical origin, time since first immigration and employment status.

## Methods

### Study group

We chose a narrow definition of the concept “immigrant” by including only legally immigrated persons born outside Sweden with both parents also born outside Sweden. Since our aim was to examine health outcomes for groups of different geographical origin, this definition is instrumental, since it only targets migrants, while excluding persons born in Sweden to immigrant parent(s). Native Swedes are defined as persons born in Sweden, with two parents born in Sweden.

The inclusion criteria for the immigrant cohorts were all persons who had immigrated legally since 1960, and who in 1990:

•were registered as residents in Sweden, and

•were between 28 and 47 years old, and

•were registered as employed or self-employed or were registered at the Public Employment Service as unemployed.

The studied outcomes were observed from 1991 to 2008. Since the main aim of the study was to examine the impact of employment/unemployment on health, we excluded all individuals who had any of the examined disorders, disability pension or death in 1990. We also chose to exclude those not registered within the national labor market system, since there were indications that a large proportion of these subjects were temporary residents in Sweden.

After these exclusions, a total of 243 860 legally immigrated persons (124 662 men, 119 198 women) were included in the study. These were compared with a random age-matched sample of 859 653 native Swedes (445 348 men, 414 305 women). Of the immigrants, 89.8% of men and 89.4% of women were employed or self-employed in 1990. Corresponding figures for native Swedes were 95.9% for men and 94.1% for women. The remainder were registered as unemployed.

### Geographical origin

The study group was classified into four sub-groups: Nordic, EU15+, Eastern Europe, and non-Europe. The first three groups consisted mainly of labor migrants and kin, while the last was dominated by refugees and kin. The characteristics of each group are as follows:

#### Nordic

The largest group had 111 073 immigrants from Denmark, Norway, Finland and Iceland (45.5% of the immigrant cohort).

#### EU 15+

This group comprised 30 660 persons (12.5% of the immigrant cohort) from Western European EU member states (apart from Nordic countries), plus Andorra, Monaco, Lichtenstein and Switzerland. The group also included immigrants from USA, Canada, Australia and New Zealand.

#### Eastern Europe

This group included 50 042 subjects from Eastern Europe, including countries of the former Soviet Union and Turkey (20.5% of the immigrant cohort). Many were labor migrants from the 1960s and early 1970s, especially from former Yugoslavia and Turkey, while others were refugees from, for example, Czechoslovakia, Poland, Bulgaria, Romania and Turkey.

#### Non-Europe

The non-European group (excluding North America and the Antipodes) consisted of 52 085 migrants (21.5% of the immigrant cohort); 42% originated from western Asia, 24% from South or Central America, 18% from central and eastern Asia, and 16% from Africa.

### Health variables studied

Only severe cases are hospitalized in Sweden, and the disorders studied were severe enough to require hospital care, and therefore likely to be good indicators of health status. The included diagnoses relate in many cases to the work environment and working conditions. Lung disorders attributed to work exposure are pneumoconiosis (mainly from silica and asbestos), lung cancer (mainly from asbestos and radon), and asthma (from dust and different chemicals) [24]. Heart disorders can be caused by some rare chemicals [25,26] and psychosocial factors, such as high demands and low control at work [27]. The role of work factors in psychiatric disorders is debated [28]. Depression is associated with traumatic life events that can also be work-related, including high stress levels as well as low control. Osteoarthritis of the hip and knee requiring hospitalization are the main musculoskeletal disorders associated with work exposures [29]. The diagnoses included in the analyses of hospitalization were from the International Classification of Diseases versions 9 and 10 (ICD 9 and ICD 10) as displayed in Table 1.

**Table 1:** Diagnoses included in the analyses of hospitalization

**Lung diseases**	ICD 9: 491, 492, 493, 495, 500, 501, 502, 503, 504, 505, 506, 507 ICD 10: J43, J44, J45, J60-J69
**Heart disorders**	ICD 9 : 401, 402, 403, 404, 413, 410, 414, 425, 428, ICD 10: I10, I11, I12, I13, I20, I21, I25, I42, I50
**Musculoskeletal disorders**	ICD 9: 715, 722, 723, 724, 726, 727, 728, 729 ICD 10: M15, M16, M17, M50, M51, M53, M54, M65, M70, M71, M75, M77, M79
**Psychiatric diseases**	ICD 9: 300, 308, 309 ICD 10: F32, F33, F34, F38, F41, F43, F45, F48

### Time factor

To examine the impact of time since first immigration, we split the immigrant cohort into two groups: those who had their immigration first recorded less than 10 years before 1990 (67%, median time was 4 years for men and 5 years for women), and those who were registered as immigrants more than 10 years before 1990 (33%, median time since first immigration was 20 years for men and 19 years for women).

### Registers used

The data supporting this study were from three national, annually updated databases: LISA (Longitudinal Integration Database for Health Insurance and Labour Market Studies), the National Patient Register, and the Cause of Death Register. These national registers are of a high quality, including personal identification numbers which enable all registered residents in Sweden to be followed in the separate registers, and through linkages between them.

LISA includes all persons aged 16 years or older from 1990 onwards. The National Patient Register includes data on hospitalizations since 1987. The Cause of Death Register includes all deceased persons registered in Sweden at the time of death from 1961, irrespective of where the death occurred.

Data on employment status, education and income in 1990, and disability pension 1991–2008 were obtained from the LISA database. The outcomes for the chosen diagnoses for 1991–2008 were collected from the National Patient Register, and the diagnoses were defined by ICD-9 and ICD-10 codes. ICD-9 was replaced by ICD-10 in 1996/1997 with slight changes within each diagnostic group. Mortality data 1991–2008 were collected from the Cause of Death Register.

### Statistical analysis

Hazard ratios (HR), with 95% confidence intervals, were calculated by Cox regression analysis in SAS 9.2 Proc Phreg (SAS Institute Inc,. Cary, NC, USA). All analyses were made for outcomes from 1991 to 2008, and separately by sex, geographical origin and employment status in 1990 (employed/self-employed or unemployed). Person-years under risk were calculated to the first event of emigration, hospitalization for the diagnoses studied, disability pension and mortality, or to the end of the study year 2008. The models were first crude and then adjusted for baseline age (continuous) as measured in 1990, and for 1990 levels of education (low, middle, high), income (continuous), and time since the first immigration, <10 or ≥10 years (Tables 2,3,4,5,6). The time factor was also analyzed separately, adjusted for the same variables as above, plus white or blue collar employment. No time-varying covariates were used.

**Table 2:** Outcomes, total immigrant cohort

	**Immigrant men, unadjusted**	**Immigrant women, unadjusted**	**Immigrant men, adjusted**	**Immigrant women, adjusted**
**Lung disorders**	1.3 (1.2-1.4)	1.2 (1.1-1.2)	1.1 (1.0-1.2)	1.1 (1.0-1.2)
**Heart disorders**	1.3 (1.3-1.4)	1.4 (1.3-1.4)	1.4 (1.3-1.4)	1.4 (1.3-1.4)
**Psychiatric dis.**	1.3 (1.2-1.3)	1.4 (1.4-1.5)	1.1 (1.1-1.2)	1.3 (1.2-1.4)
**Musculoskeletal dis.**	1.1 (1.0-1.1)	1.2 (1.1-1.2)	1.1 (1.0-1.1)	1.2 (1.1-1.2)
**Disability pension**	2.1 (2.1-2.2)	1.8 (1.8-1.8)	1.9 (1.9-2.0)	1.7 (1.7-1.7)
**Mortality**	1.3 (1.2-1.3)	1.0 (1.0-1.1)	1.2 (1.1-1.2)	1.0 (0.9-1.0)

Hazard ratios (HRs) with 95% confidence intervals (CIs) for the studied outcomes among the total immigrant cohort (124 662 men**,** 119 198 women) in relation to the Swedish control group (445348 men, 414305 women). Columns 3 and 4 are adjusted for age, educational level, income, and years since first immigration (more or less than 10 years since first immigration) as measured 1990.

**Table 3:** Employed/self-employed immigrant men

	**Lung disorder**	**Heart disorders**	**Psychiatric disorder**	**Musculo-skeletal**	**Disability pension**	**Mortality**
**Nordic**	1.4 (1.2-1.6)	1.5 (1.4-1.5)	1.2 (1.1-1.3)	1.1 (1.1-1.2)	1.7 (1.7-1.8)	1.5 (1.4-1.6)
**EU15+**	0.9 (0.7-1.1)	1.0 (1.0-1.1)	1.1 (0.9-1.2)	1.0 (0.9-1.0)	1.6 (1.5-1.6)	0.9 (0.8-0.9)
**E. Europe**	0.8 (0.7-1.0)	1.4 (1.4-1.5)	1.1 (1.0-1.2)	1.0 (0.9-1.0)	3.0 (2.9-3.1)	0.9 (0.9-1.0)
**Others**	1.3 (1.0-1.6)	1.3 (1.3-1.4)	1.1 (1.0-1.2)	0.9 (0.8-1.0)	2.3 (2.2-2.4)	0.8 (0.9-1.0)
**Total**	1.2 (1.0-1.3)	1.4 (1.3-1.4)	1.2 (1.1-1.2)	1.1 (1.0-1.1)	2.0 (1.9-2.0)	1.2 (1.2-1.2)

**Table 4:** Employed/self-employed immigrant women

	**Lung disorder**	**Heart disorders**	**Psychiatric disorder**	**Musculo-skeletal**	**Disability pension**	**Mortality**
**Nordic**	1.2 (1.1-1.3)	1.5 (1.5-1.6)	1.3 (1.2-1.4)	1.2 (1.2-1.3)	1.4 (1.4-1.5)	1.1 (1.0-1.1)
**EU15+**	0.8 (0.6-1.1)	0.9 (0.8-1.0)	1.2 (1.0-1.3)	1.0 (0.9-1.1)	1.5 (1.5-1.6)	0.8 (0.7-0.9)
**E. Europe**	0.8 (0.7-1.0)	1.3 (1.2-1.4)	1.4 (1.3-1.6)	1.2 (1.1-1.2)	2.9 (2.8-3.0)	0.9 (0.8-0.9)
**Others**	1.4 (1.1-1.8)	1.2 (1.0-1.3)	1.4 (1.2-1.6)	1.2 (1.1-1.3)	2.3 (2.2-2.4)	0.8 (0.7-0.9)
**Total**	1.1 (1.0-1.2)	1.4 (1.3-1.4)	1.3 (1.2-1.4)	1.2 (1.1-1.2)	1.7 (1.7-1.8)	1.0 (0.9-1.0)

**Table 5:** Unemployed immigrant men

	**Lung disorder**	**Heart disorders**	**Psychiatric disorder**	**Musculo-skeletal**	**Disability pension**	**Mortality**
**Nordic**	0.8 (0.5-1.3)	1.5 (1.3-1.7)	1.0 (0.8-1.3)	1.4 (1.2-1.6)	1.6 (1.4-1.7)	1.6 (1.4-1.7)
**EU15+**	0.4 (0.2-1.2)	0.8 (0.6-1.1)	0.8 (0.5-1.2)	0.9 (0.6-1.2)	1.0 (0.8-1.1)	0.5 (0.4-0.7)
**E. Europe**	0.7 (0.4-1.4)	1.2 (1.0-1.4)	0.8 (0.6-1.1)	1.0 (0.8-1.2)	1.7 (1.5-1.8)	0.5 (0.4-0.7)
**Others**	0.6 (0.3-1.1)	1.0 (0.8-1.2)	0.7 (0.5-1.0)	1.0 (0.8-1.3)	1.3 (1.2-1.5)	0.4 (0.4-0.6)
**Total**	0.7 (0.5-1.0)	1.2 (1.1-1.4)	0.9 (0.8-1.1)	1.2 (1.1-1.4)	1.4 (1.4-1.5)	1.0 (0.9-1.1)

**Table 6:** Unemployed immigrant women

	**Lung disorder**	**Heart disorders**	**Psychiatric disorder**	**Musculo-skeletal**	**Disability pension**	**Mortality**
**Nordic**	1.4 (1.0-1.9)	1.7 (1.5-2.0)	1.4 (1.2-1.7)	1.2 (1.0-1.4)	1.3 (1.3-1.4)	1.0 (0.8-1.2)
**EU15+**	1.2 (0.6-2.4)	1.2 (0.8-1.8)	0.9 (0.6-1.4)	0.8 (0.6-1.2)	1.1 (1.0-1.3)	0.8 (0.5-1.2)
**E. Europe**	0.3 (0.2-0.7)	1.0 (0.8-1.3)	1.2 (0.9-1.5)	1.0 (0.8-1.2)	1.8 (1.7-1.9)	0.6 (0.5-0.8)
**Others**	0.6 (0.3-1.2)	1.4 (1.0-1.9)	1.2 (0.9-1.6)	1.3 (1.1-1.7)	1.6 (1.5-1.8)	0.6 (0.4-0.8)
**Total**	1.1 (0.8-1.4)	1.5 (1.3-1.7)	1.3 (1.1-1.5)	1.1 (1.0-1.3)	1.4 (1.4-1.5)	0.9 (0.7-1.0)

Outcomes among employed/self-employed and unemployed immigrants from different geographical origin. HRs with 95% CIs for the studied outcomes among employed/self-employed and unemployed immigrants from different geographical origin, compared with native Swedish men and women, adjusted for age, white- and blue collar employment, educational level, income, and years since first immigration as measured 1990.

## Results

The main findings of this study are that compared to native Swedes:

•Nordic immigrants had a higher risk for most of the investigated outcomes;

•the EU15+ immigrants had an equal or lower risk of most of the investigated disorders and mortality;

•all immigrants had an elevated risk for disability pension;

•all non-Nordic immigrants had an equal or lower risk of mortality;

•unemployed non-Nordic men displayed equal or less risk than unemployed Swedes.

We also found that:

•male immigrants of more than 10 years had less risk of most examined outcomes than men with a shorter time since first recorded immigration;

•female immigrants of more than 10 years had an elevated risk of most examined outcomes than women with a shorter time since first recorded immigration.

The total immigrant cohort displayed increased HRs for almost all examined diagnoses and mortality, and pronounced higher HRs for receipt of disability pension than native Swedes. Adjustment for age, education, income, and years since first immigration, slightly lowered the immigrants’ initial HRs (Table 2).

After breaking down into geographic origin and employment status, with adjustment for age, education level, income, and years since first immigration, a more diverse pattern emerged (Tables 3,4,5,6). The Nordic group displayed higher risks for almost all the outcomes. The HR for disability pension was greater than 1.0 for all but unemployed EU15+ men, while the HRs for mortality was less than 1.0 for all except the Nordic group. Equal or lower HRs were also found among unemployed non-Nordic male immigrants for most outcomes, except disability pension.

The analyses above were adjusted for time since first immigration, but we also examined the outcomes separately in relation to this variable. When adjusted for age and employment status (blue collar, white collar or unemployed) most male groups with more than 10 years since first immigration had lower risks than males with less than 5 years since first immigration (Table 7). This is especially true for Nordic and non-European immigrants. Most female groups showed the reverse pattern, the only exception being non-European immigrants (Table 8).

**Table 7:** Men

**Men**	**Years**	**Lung disorder**	**Heart disorders**	**Musculo-skeletal**	**Psychiatric disorder**	**Disability pension**	**Mortality**
**Nordic**	< =10	1.7 (1.2-1.3)	1.5 (1.4-1.7)	1.3 (1.2-1.5)	1.6 (1.4-1.9)	1.8 (1.7-1.9)	1.9 (1.7-2.1)
	> 10	1.2 (1.1-1.3)	1.5 (1.4-1.5)	1.2 (1.1-1.2)	1.2 (1.1-1.3)	1.7 (1.7-1.7)	1.5 (1.4-1.5)
**EU15+**	< =10	1.3 (0.8-2.0)	0.9 (0.8-1.0)	1.1 (0.9-1.2)	1.0 (0.8-1.3)	1.3 (1.2-1.4)	0.9 (0.8-1.1)
	> 10	0.8 (0.6-1.0)	1.0 (1.0-1.1)	1.0 (0.9-1.0)	1.1 (1.0-1.3)	1.6 (1.5-1.7)	0.8 (0.8-0.9)
**E. Eur.**	< =10	1.0 (0.7-1.3)	1.5 (1.4-1.6)	1.0 (0.9-1.1)	1.3 (1.1-1.5)	2.7 (2.6-2.8)	0.8 (0.7-0.9)
	>10	0.8 (0.7-1.1)	1.4 (1.3-1.5)	1.0 (0.9-1.1)	1.1 (1.0-1.2)	2.9 (2.9-3.0)	1.0 (0.9-1.0)
**Non-Eur.**	< =10	1.3 (1.1-1.6)	1.3 (1.-1.4)	1.0 (0.9-1.1)	1.2 (1.1-1.3)	2.2 (2.1-2.2)	0.7 (0.6-0.8)
	>10	1.2 (0.9-1.6)	1.3 (1.-1.4)	0.8 (0.8-0.9)	1.0 (0.9-1.2)	2.0 (1.9-2.1)	0.7 (0.7-0.8)
**Total**	< =10	1.3 (1.1-1.5)	1.3 (1.-1.4)	1.1 (1.0-1.1)	1.2 (1.2-1.3)	2.1 (2.1-2.2)	0.9 (0.9-1.0)
	>10	1.1 (1.0-1.2)	1.4 (1.3-1.4)	1.1 (1.0-1.1)	1.1 (1.1-1.2)	1.9 (1.9-2.0)	1.2 (1.1-1.2)

**Table 8:** Women

**Women**	**Years**	**Lung.dis.**	**Heart disorders**	**Musculo-skeletal**	**Psych. dis.**	**Disability pension**	**Mortality**
**Nordic**	< =10	1.2 (0.9-1.6)	1.3 (1.1-1.5)	1.2 (1.1-1.3)	1.2 (1.1-1.4)	1.2 (1.1-1.3)	1.3 (1.2-1.5)
	> 10	1.2 (1.1-1.3)	1.5 (1.5-1.6)	1.2 (1.2-1.3)	1.3 (1.2-1.4)	1.4 (1.4-1.5)	1.0 (1.0-1.1)
**EU15+**	< =10	0.7 (0.4-1.3)	0.6 (0.5-0.9)	0.8 (0.7-1.0)	1.0 (0.8-1.2)	1.1 (1.0-1.2)	0.8 (0.6-1.0)
	> 10	0.9 (0.7-1.2)	1.0 (0.8-1.1)	1.0 (1.0-1.2)	1.2 (1.0-1.4)	1.6 (1.5-1.6)	0.8 (0.7-0.9)
**E. Eur.**	< =10	0.7 (0.5-1.0)	1.1 (1.0-1.3)	1.0 (0.9-1.1)	1.4 (1.2-1.6)	2.1 (2.1-2.2)	0.8 (0.7-0.9)
	>10	0.8 (0.6-1.0)	1.2 (1.1-1.3)	1.2 (1.1-1.3)	1.4 (1.3-1.5)	2.8 (2.8-2.9)	0.9 (0.8-1.0)
**Non-Eur.**	< =10	1.4 (1.1-1.7)	1.1 (1.0-1.2)	1.2 (1.1-1.3)	1.4 (1.2-1.5)	2.0 (1.9-2.0)	0.7 (0.6-0.8)
	>10	1.1 (0.8-1.5)	1.1 (1.0-1.3)	1.1 (1.0-1.3)	1.3 (1.1-1.5)	1.9 (1.8-2.0)	0.8 (0.7-1.0)
**Total**	< =10	1.1 (0.9-1.2)	1.1 (1.0-1.2)	1.1 (1.0-1.2)	1.3 (1.2-1.4)	1.8 (1.7-1.8)	0.9 (0.8-0.9)
	>10	1.1 (1.0-1.2)	1.4 (1.3-1.4)	1.2 (1.1-1.2)	1.3 (1.2-1.4)	1.7 (1.7-1.7)	1.0 (0.9-1.0)

Time since first immigration, more or less than ten years**.** HRs with 95% CIs for hospitalization for the studied outcomes for immigrants by region of origin and sex, compared with native Swedish men and women, adjusted for age and labour-market position (blue collar, white collar, unemployed) as measured 1990.

## Discussion

### Comparison with previous investigations

Our initial analysis of the total cohort showed that almost all groups had an elevated risk of all the examined diagnoses, disability pension receipt and mortality. Adjustment for age, socioeconomic factors and time since first immigration did not markedly influence the outcomes. A decrease in adjusted HRs compared with crude HRs was found in a study on immigrants in Sweden [30]. It was suggested that the higher unadjusted values can be interpreted as resulting from the immigrants’ experiences and situation in Sweden rather than their experiences before migration. Similar results from other studies have been reported, and one theory is that there is a selection of healthy persons who have the strength and inclination to migrate and settle in a new country, the so-called “healthy migrant effect” [31,32]. Our findings illustrate the necessity of taking variables such as geographic origin, different diagnoses, employment status, and time since immigration into account, in order to achieve a more nuanced knowledge about immigrants’ health status.

#### Geographical origin

After stratification into geographical origin and employment status, a more diversified risk pattern emerged. Only Nordic immigrants showed higher risks for all studied outcomes. An easier migration process with a more predictable outcome does not require potentially healthy migrant selection. Nordic migrants of both sexes more often occupy blue collar jobs than any other group, and may therefore be exposed to more health-damaging working conditions. They are also accustomed to similar social welfare in their native countries, which makes the Swedish systems more transparent and thereby more accessible for this group. These interpretations may partly explain the increased HRs for the Nordic immigrants, but the results for this group require further investigation.

#### Employment status

The examined population was divided into two groups regarding employment status (employed/self-employed and unemployed). The relative wellbeing of unemployed men, except the Nordic group, is puzzling. It may, however, indicate that the higher HRs for the active workforce are caused by their working conditions. It is generally assumed that unemployment increases the risk of poor health, especially psychiatric problems. This study rather points in a diverging direction, since unemployed men presented equal or lower risk of hospitalization for psychiatric disorders than Swedish unemployed men, while all but the EU15+ group among unemployed women presented with increased risk of psychiatric disorders compared with Swedish unemployed women. The possible explanation that immigrants are less likely to seek professional treatment when experiencing mental disorders is partly contradicted by the outcome for employed men and women of all origins. A possible explanation for the elevated risk among the active workforce, not explored in this study, could be the existence of harassment and discrimination towards immigrants in the workplace.

#### Hospitalization

There are studies indicating that immigrants do not seek medical treatment as often as natives because of cultural differences, distrust in Western medicine, or discrimination. This might partially explain low ratios for immigrants compared with Swedes. However, the studied disorders are severe, and normally lead to hospitalization (except musculoskeletal disorders). There are no economic barriers for healthcare utilization among legal immigrants in Sweden, since it is free of charge to everybody regardless of income or other selection criteria.

##### Heart disorders

Most groups displayed HRs >1. A study on Swedish residents was consistent with these findings, and also showed that many immigrant groups had a high prevalence of risk factors for coronary diseases compared with native Swedes, such as smoking, inactivity and obesity [33]. A recent Swedish study on inhabitants in Malmö (n=114 917, 15.2% born outside Sweden) confirmed our findings of increased risk of hospitalization for heart failure among immigrants from Finland and Eastern Europe [34].

In studies on Finnish male twin pairs where one twin had moved to Sweden, cardiovascular functions were better in the twin in Sweden compared with the one who stayed in Finland [17,18]. Sweden has a lower heart disorder incidence than Finland and the results may indicate different lifestyle factors influencing heart disorders.

Another study investigated the mortality from heart disorders in different immigrant groups in Sweden [35]. Finnish women showed a significantly higher risk of death from circulatory disease and ischemic heart disorder, as did Eastern European women. These findings are supported by our study. The same researchers also compared differences in coronary heart morbidity and mortality between immigrants to Sweden and residents in their countries of origin [36,37]. Differences in both directions were found among immigrants from different origins.

##### Psychiatric disorders

For psychiatric disorders the pattern was similar, with high HRs for almost all groups, except surprisingly low risks for unemployed men. In a cross-sectional study an association between immigrants’ low social status and mental illness was found, after controlling for education, employment status, income, economic security, and social support [38]. Studies in Norway found that the level of psychological distress was significantly higher in immigrants from low and middle income countries than in native Norwegians and immigrants from high income countries. The differences between the groups were explained by negative life events, poorer somatic health, difficult economic situation, and lack of social support in poorer migrants. The post-migration situation seemed more important for psychological distress than pre-migration experiences [39,40]. These findings are partly contradicted by the outcomes for the Nordic group and unemployed men in our study.

##### Musculoskeletal disorders

Slightly elevated HRs, especially for Nordic immigrants, were found. We did not find any studies on immigrants *versus* natives regarding musculoskeletal diseases. From other descriptive studies we know that the incidence of osteoarthritis of the hip differs among different ethnic groups, probably due to hereditary factors [41].

##### Lung disorders

Nordic and non-European immigrants showed HRs >1 for hospitalization for lung disorders. The only comparative studies found between immigrants and natives for lung disorders were attributable to cancer, infectious diseases or earlier exposure to dust, which were not examined in this study.

#### Disability pension

The eligibility rules for disability pensions are equal for all subjects legally residing in Sweden, natives and migrants alike. The disability pension is related to the individual’s work capacity, and thereby directly linked to health status. This study showed an incongruous relationship between low risk of mortality, moderate risk of hospitalization and significantly increased risk of receipt of a disability pension among immigrants. However, most disorders leading to a disability pension do not call for hospitalization, and a study of all diagnoses resulting in a disability pension would be of interest.

In one of the few studies identified, immigrants in Sweden had, in general, a higher risk of receiving a disability pension than natives [42]. Another study found that receipt of a disability pension differed according to country of birth. For example, it was three times more common among Finns and over five times more frequent among Greeks than among native Swedes. Possible causes for the differences could not be identified, although education, country of birth and marital status appeared to be important factors [43]*.* Another study examined the risk factors for receipt of a disability pension in a population-based cohort of individuals on long-term sick leave. It found the most important factors to be higher age, low income, previous sick leave, unemployment and non-Swedish origin (odds ratio (OR) 1.3 for men, and 1.5 for women) [44].

A multilevel analysis of the entire population aged 40–64 years in 2003 in Malmö, Sweden, found that persons originating from middle income countries (World Bank Classification of Country Economies) had the highest likelihood of receiving a disability pension [45]. A study on immigrants in Norway, contradicted our result. It followed up a large health survey, performed in 2000–2001, for receipt of disability pensions 4 years later. The age- and gender-adjusted OR was 2.3. However, when adjusting for occupation, working conditions and income, the OR was reduced to 0.9 [46].

A study on immigrants in the Netherlands observed that consulting physicians differentiated between native and immigrant patients. The ethnic categorization by the physicians triggered an interpretation of the immigrant patients’ behavior in cultural terms, which reduced the physicians’ capacities to adapt their consulting activities to the needs of the immigrants. The result was that many immigrants remained work-incapacitated for longer periods, with higher risk of ending up in the disability pension program [47].

#### Mortality

There has been an interest for some decades in mortality studies of immigrant populations. In 1984, Marmot reported the results of a systematic review of mortality among immigrant groups in England and Wales. Mortality rates in 1970–1972 were compared with rates in the immigrants’ countries of origin. All-cause male mortality was lower in immigrants from Italy, the Caribbean, and Poland than in the countries of origin, suggesting a selective effect among migrants. The opposite pattern applied for immigrants from Ireland, suggesting that, because of the short geographical distance and cultural similarities, social and health disadvantages may be a stimulus to migration [48]. If so, this could partly explain the increased mortality risk for the Nordic immigrants in our study.

A Swedish register study 1991–1998 reported that all-cause mortality was lower for immigrants to Sweden compared with all-cause mortality in their country of birth [36], which may indicate a healthy migrant effect, or/and that migrants acquire improved living and health conditions in Sweden. A study of mortality patterns among Canadian immigrants, refugees and non-refugees, 1980–1998, found that immigrants presented lower all-cause mortality compared with the general Canadian population (standardized mortality ratio between 0.34 and 0.58). Mortality rates differed by region of birth and were higher among refugees than other immigrants [49].

The low mortality risk for all groups in this study, except the Nordic group, whether employed or unemployed, is striking. This might be explained by the “healthy migrant effect”, i.e., select persons with a more favorable life prognosis. Another proposed explanation is that the lower mortality rate of migrants is a denominator effect, caused by immigrants leaving the country without reporting to the authorities, hence their deaths are not recorded [50,51].

#### Time factor

Studies on migrants’ health changes over time often refer to acculturation (e.g., occupational status, language skills, social and cultural integration) as an active factor causing differences between immigrant groups and between immigrants and the native population. Although the definition of the concept is much discussed [52,53], it has been instrumental in explaining the more favorable health status of minorities compared with natives [54], as well as the opposite [55], while other studies found more heterogeneous outcomes [56]. If acculturation is a factor in this study, it tends to agree with the latter, since there are contradictory outcomes, such as the favorable health outcomes for unemployed non-Nordic men (Table 5), and the improved health over time for most male groups, contrasted by the higher risks for most female groups with a longer period of residence (Tables 7, 8).

#### Methodological consideration

This study covers documented immigrants and Swedish-born persons aged 28 to 47 in 1990, who were followed in registers from 1991 to 2008. We chose the age of 28 at the lower end of the age spectrum as the baseline in the year 1990 for two reasons: the Swedish social security system has different sets of rules for persons with reduced work capacity younger and older than 28, and it is also a reasonable age to be established in the labor market. The upper end of the age spectrum was 47, as these persons would reach 65, the common age for retirement, in 2008. The studied disorders can, of course, appear later in life, but we do not believe that the distribution among groups of different geographical origins would change markedly.

The strengths of the study are that it included all immigrants matching the selection criteria, and that the Swedish registers are of a high quality, based on personal identification numbers connected to the country of birth, which enables all registered residents in Sweden to be followed in separate as well as in linked registers. Since the aim of the study was to investigate health outcomes for immigrants compared with native Swedes, we decided to adjust for socioeconomic status, measured as education level and income. This study can only offer a limited contribution to the understanding of health, disability pension receipt and mortality among immigrants compared with native Swedes, since the data do not allow us to identify all confounders of the outcomes, e.g., lifestyle, medical history, and co-morbidity not resulting in hospitalization. Some data, such as education level, are self-reported, which can result in underestimation as well as overestimation of the effects of education. We must also point out that we examined only a selection of the four main ICD diagnostic groups. The chosen diagnoses were, however, those known to be potentially work-related. Other studies have shown that migrants have elevated mortality risks and hence higher risks for other disorders, e.g., stroke, diabetes, infectious diseases and certain forms of cancer [42].

For this study, geographical areas were merged and the outcomes could differ from one country to another within each of the four groups, and would not be detected here.

Employment status and income were registered in 1990. Many individuals have certainly changed their socioeconomic status during follow-up. This is especially probable for young persons who were unemployed in 1990. Changes in occupational status, exposure patterns and socioeconomic position since 1990 could therefore have had an impact on health that is not revealed here. The rough division into white and blue collar work in 1990 is approximate and more detailed information on occupation can give more precise information about the health status of the population. It must also be mentioned that references to previous studies suffer from inconsistencies, since crucial concepts, such as “immigrant” and “native”, are defined differently in different studies.

## Conclusion

This study shows that, when the data were adjusted for age, educational level, income and years in Sweden, almost all foreign-born groups, except the Nordic group, have outcomes which are mostly equal to, or more favorable than, the native population regarding the examined diagnoses and mortality. However, all groups of immigrants had increased risk of receipt of a disability pension. The reason for this ought to be examined more thoroughly, both from a medical point of view, and in the context of the labor market and social security policies. The favorable results for unemployed non-Nordic men also encourage further studies. Future investigations should also seek to determine if the low mortality risk for many immigrant groups is a “healthy migrant effect”, a denominator problem, or both. Knowledge of work-related migrant health is very fragmentary. It is probable that migrants will form an increased proportion of the workforce in Sweden and many other European countries, because of the demographic structure in the region. It is therefore of great importance to address these issues.

## Competing interests

The authors declare that they have no competing interests.

## Authors’ contributions

BJ has as main author made substantial contributions to the conception, design and interpretation of the data. MH, IL, TN, OL, PL, and EV have all been involved in designing the study, drafting the manuscript, or revising it critically for important intellectual content. TN performed the statistical analyses. All authors read and approved the final manuscript.

## Pre-publication history

The pre-publication history for this paper can be accessed here:

http://www.biomedcentral.com/1471-2458/12/845/prepub
